# Silibinin as potential tool against SARS‐Cov‐2: In silico spike receptor‐binding domain and main protease molecular docking analysis, and in vitro endothelial protective effects

**DOI:** 10.1002/ptr.7107

**Published:** 2021-04-06

**Authors:** Antonio Speciale, Claudia Muscarà, Maria Sofia Molonia, Francesco Cimino, Antonella Saija, Salvatore Vincenzo Giofrè

**Affiliations:** ^1^ Dipartimento di Scienze Chimiche, Biologiche, Farmaceutiche ed Ambientali Università di Messina Messina Italy

**Keywords:** docking, endothelial dysfunction, protease, SARS‐CoV‐2, silibinin, spike protein

## Abstract

The spread of SARS‐CoV‐2, along with the lack of targeted medicaments, encouraged research of existing drugs for repurposing. The rapid response to SARS‐CoV‐2 infection comprises a complex interaction of cytokine storm, endothelial dysfunction, inflammation, and pathologic coagulation. Thus, active molecules targeting multiple steps in SARS‐CoV‐2 lifecycle are highly wanted. Herein we explored the in silico capability of silibinin from *Silybum marianum* to interact with the SARS‐CoV‐2 main target proteins, and the in vitro effects against cytokine‐induced‐inflammation and dysfunction in human umbilical vein endothelial cells (HUVECs). Computational analysis revealed that silibinin forms a stable complex with SARS‐CoV‐2 spike protein RBD, has good negative binding affinity with Mpro, and interacts with many residues on the active site of Mpro, thus supporting its potentiality in inhibiting viral entry and replication. Moreover, HUVECs pretreatment with silibinin reduced TNF‐α‐induced gene expression of the proinflammatory genes IL‐6 and MCP‐1, as well as of PAI‐1, a critical factor in coagulopathy and thrombosis, and of ET‐1, a peptide involved in hemostatic vasoconstriction. Then, due to endothelium antiinflammatory and anticoagulant properties of silibinin and its capability to interact with SARS‐CoV‐2 main target proteins demonstrated herein, silibinin could be a strong candidate for COVID‐19 management from a multitarget perspective.

## 1 INTRODUCTION

Starting from December 2019 the world has faced an extraordinary health emergency caused by the new strain of Coronavirus, called SARS‐CoV‐2, responsible for the coronavirus disease 2019 (COVID‐19). After a rapid worldwide spread of the disease, in March 2020, the World Health Organization (WHO) declared COVID‐19 outbreak as pandemic. February 2021 counted a total of more than 110 million confirmed cases globally and more than 2 million deaths, reaching all countries worldwide [WHO Health Emergency Dashboard https://covid19.who.int Date: 2021 Date accessed: February 25, 2021]. In mild cases, SARS‐CoV‐2 infection shows fever, fatigue, and dry cough, while in severe cases pneumonia, respiratory, and kidney failure was observed. In addition, this infection may be complicated by lymphopenia and interstitial pneumonia with high levels of proinflammatory mediators inducing a cytokine storm which, in turn, can produce acute respiratory distress syndrome (ARDS), organ failure, and sepsis, potentially progressing to patient's death (Zhou et al., 2020). Interestingly, a larger than expected number of thrombotic events were reported in COVID‐19 patients (Ahmed, Zimba, & Gasparyan, 2020), and elevated plasminogen active inhibitor‐1 (PAI‐1) is an independent risk factor for poor ARDS outcomes.

Presently, even if the new vaccines will prevent people from acquiring the infection and probably reduce transmission, there is no specific antiviral therapy for COVID‐19. Indeed, in current emergent situation, the concept of drug repositioning might be a cost‐effective and time‐efficient option for the development of possible therapeutic and/or prophylactic lead candidates from well‐known traditional and/or approved drugs. An ideal therapeutic approach to manage COVID‐19 would include a drug capable to directly targeting the key molecular machinery driving the virus lifecycle while at the same time preventing the clinically significant effects associated to poor prognosis such as cytokine storm and hypercoagulability.

Silymarin is a mixture of flavonolignans extracted from the milk thistle (*Silybum marianum* Gaertneri), exhibiting potent antiinflammatory and antioxidative properties (Vargas‐Mendoza et al., 2014). Silymarin, employed as standardized milk thistle extracts made from the fruits containing 30–65% silymarin as active ingredient, has become a frequently applied therapy for various liver disorders, and it is classified by the WHO Anatomical Therapeutic Chemical (ATC) classification system as liver therapy (A05BA03). Silibinin, a mixture of two stereoisomers, silibinin A and silibinin B in equimolar ratio, is the major component of this complex extract (about 60–70%), and it is biologically the most active constituent of silymarin, being so widely used in many liver diseases. In particular, silibinin reduces viral infection in patients with chronic hepatitis C (Blaising et al., 2013; Ferenci et al., 2008; Hawke et al., 2010). In fact, silibinin hinders hepatitis C virus (HCV) entry by slowing down trafficking through clathrin‐coated pits and vesicles, and so inhibiting the clathrin endocytic pathway. Thanks to this specific inhibition mechanism, silibinin could inhibit infection by other viruses that enter cells by clathrin‐mediated endocytosis, including reovirus, vesicular stomatitis, and influenza viruses, and the last discovered SARS‐CoV‐2 (Bayati, Kumar, Francis, & McPherson, 2020). Furthermore, silibinin can affect viral RNA‐dependent RNA polymerase (RdRp) activity so inhibiting HCV replication (Ahmed‐Belkacem et al., 2010). Silibinin has been reported for its antiinflammatory activity (Trappoliere et al., 2009) and protective effects against endothelial dysfunction both in vivo in db/db mice (Li Volti et al., 2011) and in vitro (Rezabakhsh et al., 2018), so indicating additional targets useful in COVID‐19 disease.

Based on the effects against HCV RNA virus and endothelial dysfunction, in this paper we explored the capability of silibinin to interact with the SARS‐CoV‐2 main target proteins and to protect against cytokine‐induced‐inflammation and dysfunction in vessel endothelial cells, so achieving multiple targeting effects for treatment or prevention of COVID‐19.

## 2 MATERIALS AND METHODS

### 2.1 In silico molecular docking experiments

The crystallographic structures of target proteins by Protein Data Bank (PDB) database were obtained. Several crystallographic structures of SARS‐CoV‐2 receptors were present in PDB and priority was given to proteins published in 2020 and with higher resolutions. Two different targets to docking studies were selected, the crystal structure of spike RBD bound with ACE2 (6M0J) which possess a high resolution of 2.45 Å, containing a total of 791 residues and 2 protein chains, and also the crystal structure of Mpro in complex with the inhibitor N3. Two crystallographic structures were used for the latter target, 6LU7 which possess a resolution of 2.16 Å containing 312 residue, and 7BQY published more recently by the same authors as the previous one, which has a high resolution of 1.70 Å containing a total of 307 residues (Jin et al., 2020).

Structure of silibinin was generated using ChemOffice v12.0 Ultra software package and has been MM2‐optimized. Before starting the docking evaluations, all water was removed, ACE2 receptor in 6M0J was removed, the partial atomic charges (Gasteiger‐Marsili formalism), as well as all the possible rotable bonds of the selected phytochemicals and the Kollman charges for all the atoms in enzymes were assigned by using the AutoDock Tools 1.5.6 version. Moreover, missing residues were also built and hydrogen atoms were added to the amino acids of the protein with the mentioned program. A receptor grid box was generated by AutoGrid4.2 centered on entire protein with grid box dimensions of *x* = 126, *y* = 126, *z* = 126, and binding radius = 0.375 Å, with absolute coordinates of grid box *x* = −37.17; *y* = 30.2; *z* = 17.072 for spike protein. Grid box for 6LU7‐MPro and 7BQY‐MPro was generated by AutoGrid4 centered on ligand with grid box dimensions of *x* = 70, *y* = 70, *z* = 70, and binding radius = 0.375 Å, with absolute coordinates of grid box *x* = −11.663, *y* = 11.606, *z* = 69.273, and *x* = 10.398, *y* = −1.254, *z* = 23.473, respectively.

Lamarckian Genetic Algorithm (LGA) implemented in AutoDock4.2 was adopted to perform docking simulations, and each docking experiment consisted of 100 docking runs with 150 individuals and 2.5 × 10^5^ energy evaluations. Other parameters were left to their default values. The docking results were used to generate inhibitor thermodynamic properties, such as free energy of binding (Δ*G*), Vdw + Hbond + desolv energy (kcal/mol), intermolecular energy (kcal/mol), and inhibition constants (Ki). The cluster with the lowest free energy of binding was visually analyzed using Biovia Discovery Studio 2017 R2 and LigandScout 2.01.

### 2.2 In silico pharmacokinetic screening of silibinin

The physicochemical properties according to Lipinski's rule were calculated for silibinin to predict the pharmacokinetics property using the online tool molinspiration (https://www.molinspiration.com/cgi-bin/properties), where the calculation of LogP is based on the formula satisfying lipophilicity, hydrophobicity, and polarity of the compound (Kujawski, Popielarska, Myka, DrabjDska, & Bernard, 2012).

Pharmacokinetic properties such as absorption, distribution, metabolism, and excretion (ADME) profiling of silibinin were determined using the online tool (http://biosig.unimelb.edu.au/pkcsm/prediction). The absorption of drugs depends on factors including membrane permeability [reported as colon cancer cell line (Caco‐2) permeability], intestinal absorption, skin permeability levels, P‐glycoprotein substrate, or inhibitor. The distribution of drugs depends on factors that include the blood–brain barrier (logBB), CNS permeability, and the volume of distribution (VDss). Metabolism is predicted based on the CYP models for substrate (CYP2D6 and CYP3A4). Excretion is predicted based on the total clearance model (Pires, Blundell, & Ascher, 2015).

### 2.3 In vitro experiments on human endothelial cells

#### 2.3.1 Reagents

Medium 199, fetal bovine serum (FBS), l‐glutamine, HEPES buffer, penicillin/streptomycin, endothelial cell growth factor, heparin, gelatin, dimethyl sulfoxide (DMSO), Dulbecco's phosphate‐buffered solution (DPBS), M‐MLV Reverse Transcriptase, RNase Inhibitor, dNTP mix, Oligo(dT)_23_, and SYBR green JumpStart Taq Ready Mix were purchased from Sigma Aldrich (Milan, Italy). Recombinant Human Tumor Necrosis Factor‐α (TNF‐α) was purchased from PeproTech, Inc. (Rocky Hill, NJ, USA). The E.Z.N.A Total RNA kit was purchased from OMEGA Bio‐tek, Inc. (Norcross, GA, USA). Qubit RNA assay kit and PCR primers were from Invitrogen (Thermo Fisher Scientific Inc., Waltham, MA, USA).

#### 2.3.2 Cell culture and treatment

Human umbilical vein endothelial cells (HUVECs) were isolated from freshly obtained human umbilical cords by collagenase digestion of the interior of the umbilical vein, and were cultured in medium 199, supplemented with 20% FBS, 1% l‐glutamine, 20 mM HEPES buffer, 100 units/mL penicillin/streptomycin, 50 mg/mL endothelial cell growth factor, and 10 μg/mL heparin, in gelatin pretreated flasks. Cells were maintained in an incubator with humidified atmosphere containing 5% CO_2_ at 37°C. Cells used in this study were from the second to fourth passage.

For all the experiments, silibinin was always freshly dissolved in DMSO and immediately used. The final concentration of DMSO in the culture medium during the different treatments was <0.1% v/v. The subconfluent cells were treated for 24 h with silibinin (range: 5–25 μM), whereas control cells were treated with 0.01% v/v DMSO only. After this incubation time, cells were washed with DPBS and then exposed for 2 h to 20 ng/mL TNF‐α.

#### 2.3.3 Quantitative RT‐PCR

Total cellular RNA was isolated with E.Z.N.A® Total RNA kit according to manufacturer's instruction, quantified using Qubit RNA assay kit, and reverse transcripted with M‐MLV Reverse Transcriptase.

For cell gene expression, mRNA levels were determined by Real‐Time qPCR (Applied Biosystems 7300 Real‐Time PGR System, GA, USA) with SYBR green chemistry (SYBR green JumpStart Taq Ready Mix), and using the following sets of primers: Endothelin‐1 (ET‐1), FW 5′‐AGAGTGTCTACTTCTGCCA‐3′, RV 5′‐CTTCCAAGTCCATACGGAACAA‐3′; Plasminogen activator inhibitor‐1 (PAI‐1), FW 5′‐ACCGCAACGTGGTTTTCTCA‐3′, RV 5′‐TTGAATCCCATAGCTGCTTGAAT‐3′; IL‐6, FW 5′‐ACTCACCTCTTCAGAACGAATTG‐3′, RV 5′‐CCATCTTTGGAAGGTTCAGGTTG‐3′; Monocyte chemotactic protein‐1 (MCP‐1), FW 5′‐CAGCCAGATGCAATCAATGCC‐3′, RV 5′‐TGGAATCCTGAACCCACTTCT‐3′ (Primer Bank ID: 4506841a1). Glyceraldehyde‐3‐phosphate dehydrogenase (GAPDH) was chosen as housekeeping gene since it showed low variability in expression levels between the different treatments (GAPDH primers: FW 5′‐GGC TCT CCA GAA CAT CAT CCC TGC‐3′, RV 5′‐GGG TGT CGC TGT TGA AGT CAG AGG‐3′). Data were elaborated by SDS 1.3.1 software (Applied Biosystems, Foster City, CA, USA) and expressed as threshold cycle (C_t_). The fold increase in mRNA expression compared with the control cells not treated and not exposed to TNF‐α was determined using the 2^‐ΔΔCt^ method (Livak & Schmittgen, 2001).

#### 2.3.4 Statistical analysis

All the experiments were performed in triplicate and repeated three times. Results are expressed as mean ± SD from three experiments and statistically analyzed by a one‐way or a two‐way ANOVA test, followed by Tukey's HSD, using the statistical software ezANOVA (http://www.sph.sc.edu/comd/rorden/ezanova/home.html). Differences in groups and treatments were considered significant for *p* < .05.

## 3 RESULTS

### 3.1 Interactions of silibinin with SARS‐CoV‐2 spike protein (6M0J)

The molecular docking analysis results for silibinin against 6M0J, including binding energy/Gibbs Energy, ligand efficiency, intermolecular energy, and van der Waals (VDW)‐H Bond desolvation energy are represented in Table 1. Silibinin showed a high negative binding affinity of −8.97 (kcal/mol) and produced great intermolecular energy and van der Waals (VDW)‐H Bond desolvation energy of −10.16 and − 10.13 (kcal/mol), respectively.

**TABLE 1:** Molecular docking analysis results for silibinin against 6M0J, including binding energy/Gibbs Energy, intermolecular energy, and van der Waals (VDW)‐H Bond desolvation energy

Compound	No‐RBD binding free energy (kcal/Mol)	RBD binding free energy (kcal/Mol)	Vdw + Hbond + desolv energy (kcal/Mol)	Intermolecular energy (kcal/Mol)
Silibinin	‐	−8.97	−10.13	−10.16

Recently, crucial residues involved in hydrogen bonding ACE2‐SARS‐CoV‐2 spike complex have been identified. In particular, the SARS‐CoV‐2 spike protein was found binding to ACE2 receptor with 11 hydrogen bonds and 1 salt bridge. Very important interactions based on structure analysis for the binding of SARS‐CoV‐2 to hACE2 have been reported and include Glu35, Tyr83, Asp38, Lys31, Glu37, His34 amino acid residues of ACE2 receptor, and Gln493, Gln498, Asn487, Tyr505, Lys417, Thr500, Tyr489, Asn501, Tyr453, and Ala475 residues in SARS‐CoV‐2 S‐protein RBD (Veeramachaneni, Thunuguntla, Bobbillapati, & Bondili, 2020). Moreover, when amino acid residues (Leu455, Phe456, Ser459, Gln474, Ala475, Phe486, Phe490, Gln493, and Phe499) were mutated in SARS‐CoV‐2, their binding affinity for ACE2 was abolished, indicating that these residues are very important for the binding of SARS‐CoV‐2 to ACE2 (Yi et al., 2020).

**FIGURE 1:**
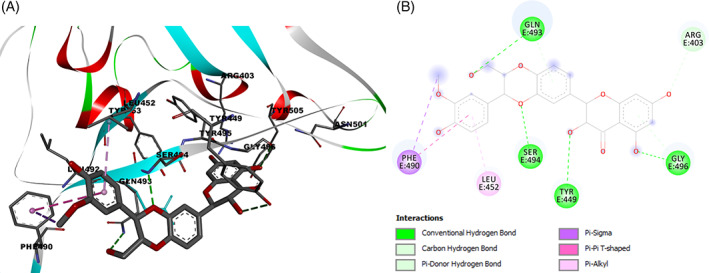
Silibinin docked in SARS‐CoV‐2 spike receptor‐binding domain bound with ACE2 (6M0J). (A) Amino acid residues involved in interaction, and (B) binding interaction of silibinin with amino acid with hydrogen For Peer Review bond (green dash line) [Colour figure can be viewed at wileyonlinelibrary.com]

On the basis of these considerations, the interactions of silibinin with the amino acid residues of the SARS‐CoV‐2 spike protein RBD site were evaluated. Results demonstrated a high affinity interaction (−8.97 kcal/mol) of silibinin with the RBD‐bound ACE2 by four hydrogen bonding between the oxygen of the hydroxymethyl group of silibinin with key residue Gln493, the oxygen of the phenolic group with Gly496, and the oxygen of the dioxane group with Ser494. The benzene ring shows pi‐pi and pi‐alkyl interaction with Phe490 and Leu452 (Figure 1). Furthermore, the best docking pose of silibinin, in ACE2‐SARS‐CoV‐2 spike complex, was found in close contact with the amino acid residues of the ACE2 receptor Lys353 and with Glu35 and Asp38, which represent some key amino acids involved in the binding of protein SARS‐CoV‐2 RBD. This analysis suggests that binding of spike protein RDB with silibinin may prevent binding to the ACE2 receptor.

### 3.2 Interactions of silibinin with COVID‐19 main protease in complex with an inhibitor N3 (6LU7‐7BQY)

Two crystallographic structures of 3Clike protease of SARS‐CoV‐2 in complex with a peptide‐like inhibitor were used: 6LU7 which possess a resolution of 2.16 Å, and 7BQY which has a high resolution of 1.70 Å. Results are summarized in Table 2. The enzyme active site is formed by His41, Met 49, Phe140, Lue141, Asn142, Gly143, His163, His164, Met165, Glu166, Leu167, Pro168, His172, Gln189, Thr190, and Ala191. The aminoacid residues His41 and Cys145 were reported as the catalytic dyad in the active site (Tahir Ul Qamar, Alqahtani, Alamri, & Chen, 2020), His164 is essential for enzyme activity. His163, His172, and Glu166 are believed to provide the opening gate for the substrate in the active state of the protomer (Yang et al., 2003), whereas Thr24, Thr26, and Asn119 are predicted to play roles in drug interactions (Liu & Wang, 2020). All of these residues play a significant role in replication and are essential for the survival of SARS‐CoV‐2 (Khan, Zia, Ashraf, Uddin, & Ul‐Haq, 2020). About that, the best docking poses of silibinin were evaluated to determine the interactions with these crucial aminoacidic residues.

**TABLE 2:** Molecular docking analysis results for silibinin against 6LU7 and 7BQY, including binding energy/Gibbs Energy, intermolecular energy, van der Waals (VDW)‐H Bond desolvation energy, and estimated inhibition constant

Compound	6LU7/7BQY Binding free energy (kcal/mol)	Vdw + Hbond + desolv energy (kcal/mol)^a^	Intermolecular energy (kcal/mol)^a^	Estimated inhibition constant, Ki (nM)^a^
Native ligand	−10.79/−9.82	−16.38	−16.46	12.25
Silibinin	−9.38/−10.17	−11.36	−11.36	35.07

^a^ Values referred to the best binding energy.

Docking protocol was validated by redocking method of cocrystallized structure in the binding site to determine the lowest RMSD relative to the crystallographic pose. Native ligand N3, was successfully redocked with a RMSD of 1.42 Å and the best pose, in complex with 6LU7, showed hydrogen bonds and interactions with the target enzyme aminoacidic residues likewise the cocrystallized structure. The successful docking protocol was employed for docking analysis of silibinin showing good negative binding affinity −10.17 (kcal/mol) and Ki = 35.07 nM and formation of five hydrogen bonds particularly with key residues His164 and Glu166, and also hydrophobic interaction with His41 in the catalytic site was found (Table 3).

**TABLE 3:** Summary of top ranked phytochemicals screened against SARS‐CoV‐2 3CLpro receptor binding site (6LU7 and 7BQY) with their respective structures, binding affinity, and interacting residues [Colour table can be viewed at wileyonlinelibrary.com]

Compound	Molecular structure and interaction with enzyme active site	Residues interacting with phytochemical compounds and binding free energy
Native ligand	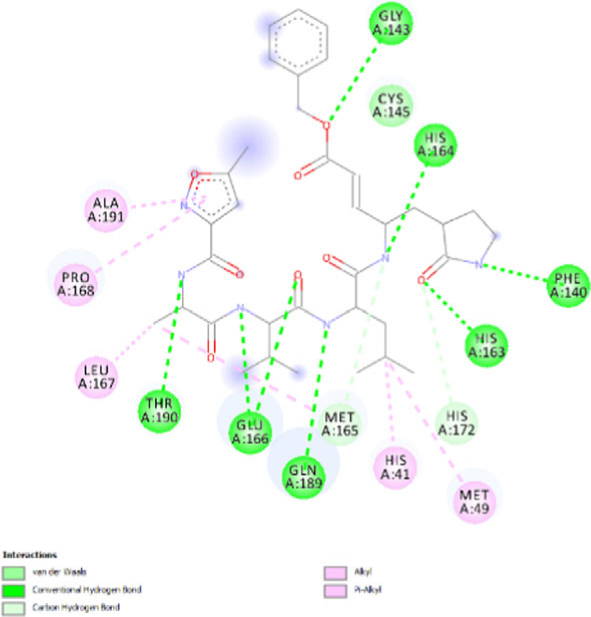	*Interactions*: Met49, **His41**, Leu167, Pro168, Ala191, Met165, His172, Met165, **Cys145**, Gly143, **His164**, Phe140, **His163**, Gln189, **Glu16**6, Thr190. −10.79 kcal/Mol
Silibinin	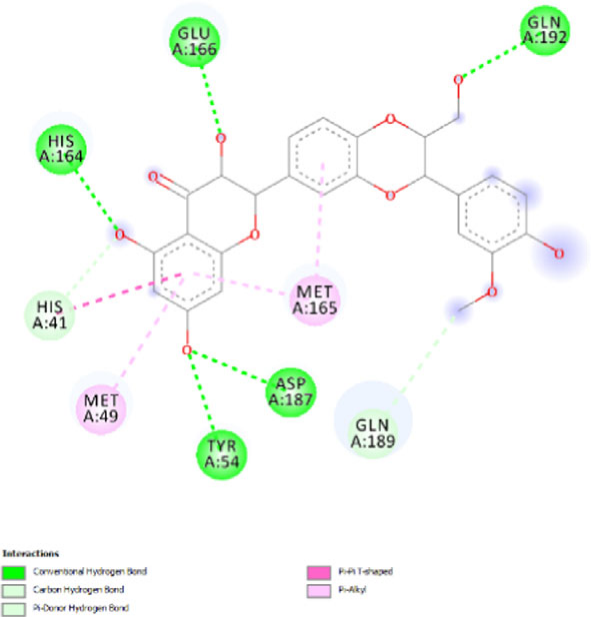	*Interactions*: Met49, Met165, **His41**, Gln189, Gln192, **Glu166**, **His164**, Tyr54, Asp187. −10.17 kcal/Mol

### 3.3 Silibinin inhibits TNF‐α‐induced proinflammatory gene expression in endothelial cells

In order to study the endothelial antiinflammatory activity of silibinin, we evaluated the transcriptional levels of MCP‐1 and IL‐6 in HUVECs exposed to TNF‐α. MCP‐1, also known as chemokine (C‐C motif) ligand 2 (CCL2), is expressed by mainly inflammatory cells and endothelial cells following proinflammatory stimuli and tissue injury, and it stimulates the migration of monocytes (Lin, Kakkar, & Lu, 2014). IL‐6 is a cytokine with pleiotropic activity not only produced by macrophages but also secreted by endothelial cells following stimulation of inflammatory cytokines such as IL‐1 or TNF‐α, and tissue hypoxia (Tanaka, Narazaki, & Kishimoto, 2016). Both MCP‐1 and IL‐6 overexpression have been implicated in several pathologic conditions including atherosclerosis, thrombosis, and inflammatory diseases (Li, Chen, Zhang, Li, & Liu, 2020; Lin et al., 2014; Oikonomou et al., 2020) and, moreover, the serum concentrations of IL‐6 and MCP‐1 in all patients with severe COVID‐19 are significantly elevated compared with those in healthy controls (Kang et al., 2020).

**FIGURE 2:**
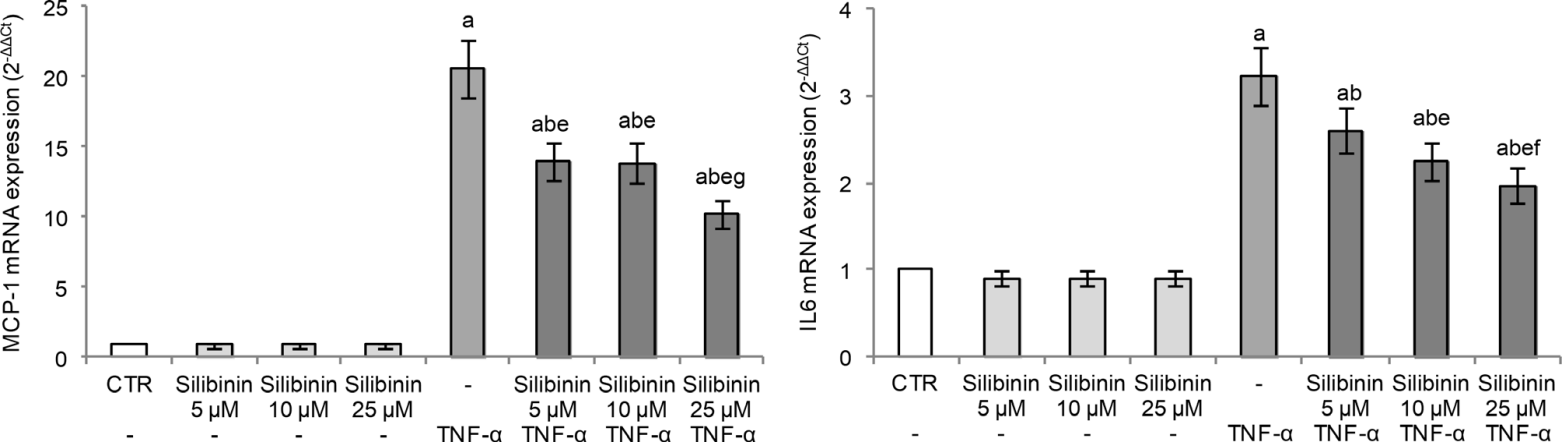
Effect of silibinin pretreatment on TNF‐α‐induced MCP‐1 and IL‐6 mRNA expression in HUVECs. Cells were pretreated or not with silibinin (range: 5–10‐25 μM), for 24 h and then exposed to 20 ng/mL TNF‐ α for 2 h. Cells treated with the vehicle alone (0.1% DMSO v/v) were used as controls (CTR). mRNA expression was analyzed by real‐time RT‐PCR and data are expressed as 2^−ΔΔCt^. GAPDH was used as housekeeping gene. Results, deriving from three independent experiments, are reported as mean ± SD. ^a^p < 0.05 versus CTR; ^b^p < 0.05 versus same concentration of silibinin alone; ^e^p < 0.05 versus TNF‐α; ^f^p < 0.05 versus silibinin 5 μM + TNF‐α; ^g^p < 0.05 versus silibinin 5 and 10 μM + TNF‐α

In our experimental conditions, TNF‐α stimulation induced an inflammatory response in endothelial cells as evidenced by the markedly increased gene expression of both MCP‐1 and IL‐6. Silibinin pretreatment effectively inhibited TNF‐α‐induced expression of these cytokines in a dose‐dependent manner, demonstrating its antiinflammatory activity in the endothelium (Figure 2). Silibinin alone was not able to affect their gene expression.

### 3.4 Silibinin inhibits TNF‐α‐induced ET‐1 and PAI‐1 expression in endothelial cells

Endothelial dysfunction is the principal determinant of microvascular disease, as it shifts the vascular equilibrium toward more vasoconstriction, causing subsequent organ ischemia, systemic inflammation with associated tissue edema, and a procoagulant state. ET‐1, the most potent vasoconstrictor in the cardiovascular system, regulates basal vascular tone and glomerular hemodynamics. Abnormal activation of the endothelin system can promote pulmonary hypertension, kidney diseases, and cardiovascular diseases, including essential hypertension, atherosclerosis, coronary artery disease, and congestive heart failure (Eroglu, Kocyigit, & Lindholm, 2020).

**FIGURE 3:**
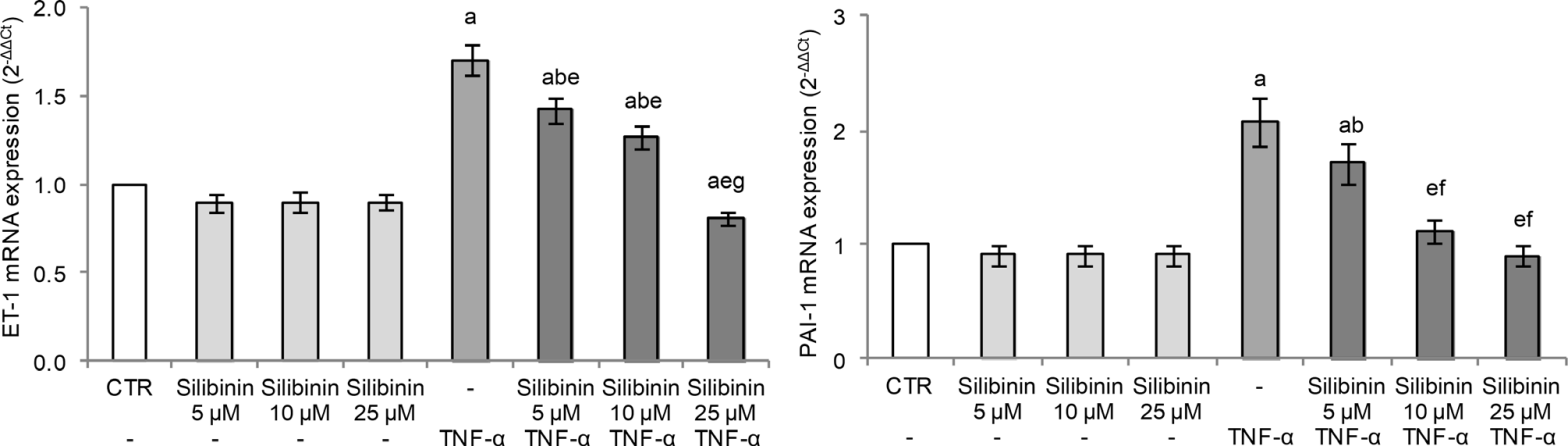
Effect of silibinin pretreatment on TNF‐α‐induced ET‐1 and PAI‐1 mRNA expression in HUVECs. Cells were pretreated or not with silibinin (range: 5–10^−25^ μM), for 24 h and then exposed to 20 ng/mL TNFα for 2 h. Cells treated with the vehicle alone (0.1% DMSO v/v) were used as controls (CTR). mRNA expression was analyzed by real‐time RT‐PCR and data are expressed as 2^−ΔΔCt^. GAPDH was used as housekeeping gene. Results, deriving from three independent experiments, are reported as mean ± SD. ^a^ *p* < .05 versus CTR; ^b^ *p* < .05 versus same concentration of silibinin alone; ^e^ *p* < .05 versus TNF‐α; ^f^ *p* < .05 versus silibinin 5 μM + TNF‐α; ^g^ *p* < .05 versus silibinin 5 and 10 μM + TNF‐α

In our experimental conditions, HUVECs exposure to TNF‐α‐induced ET‐1 gene expression (Figure 3), as elsewhere reported (Cui et al., 2018; Keiser et al., 2009). Silibinin pretreatment showed a dose‐dependent protective effect with a marked reduction of TNF‐α‐induced ET‐1 expression, with the cells treated with the higher tested silibinin concentration (25 μM) showing mRNA levels similar to the control (Figure 3).

PAI‐1 plays an important role in the regulation of fibrinolysis, as it is a plasma inhibitor of enzymes involved in this process, including urokinase (uPA) and tissue plasminogen activator (tPA). Elevated PAI‐1 levels exacerbate the progression of systemic inflammation, especially sepsis‐induced disseminated intravascular coagulation (Madoiwa et al., 2006).

TNF‐α exposure induced PAI‐1 gene expression in HUVECs (Figure 3) demonstrating altered endothelial fibrinolytic system. Silibinin pretreatment dose‐dependently protected HUVECs with a marked reduction of TNF‐α‐induced PAI‐1 expression. Interestingly, cells treated with 10 μM silibinin restored the regulatory mechanism of PAI‐1 with mRNA levels similar to the control cells (Figure 3).

### 3.5 Drug‐likeness and ADME prediction for silibinin

Since information regarding pharmacokinetic properties of a molecule are important in order to support its clinical use, in silico ADME prediction was performed using Molinspiration and pkCSM online property calculation toolkits. Results are reported in Table 4.

**TABLE 4:** Prediction of pharmacokinetic properties for silibinin

Descriptor	Unit	Predicted value
Molecular weight		482.441
miLopP		1.47
Total polar surface area (TPSA)		155.15
Rotatable bonds		4
Acceptors		10
Donors		5
Surface area		198.685
Water solubility	*Numeric (log Mol/L)*	−3.204
Caco2 permeability	*Numeric (log Papp in 10* ^*−6*^ *cm/s)*	0.435
Intestinal absorption (human)	*Numeric (% absorbed)*	61.861
Skin permeability	*Numeric (log Kp)*	−2.735
P‐glycoprotein substrate	*Categorical (yes/no)*	Yes
P‐glycoprotein I inhibitor	*Categorical (yes/no)*	Yes
P‐glycoprotein II inhibitor	*Categorical (yes/no)*	Yes
VDss (human)	*Numeric (log L/kg)*	0.369
Fraction unbound (human)	*Numeric (Fu)*	0
BBB permeability	*Numeric (log BB)*	−1.207
CNS permeability	*Numeric (log PS)*	−3.639
CYP2D6 substrate	*Categorical (yes/no)*	No
CYP3A4 substrate	*Categorical (yes/no)*	No
Total clearance	*Numeric (log ml/min/kg)*	−0.103

The Rule of Five (Ro5) or Lipinski's rule is able to predict absorption or permeation of a potential drug candidate combining specific parameters (Lipinski, Lombardo, Dominy, & Feeney, 1997). According to this, poor oral bioavailability is more likely when there are more than 5 H‐bond donors, 10 H‐bond acceptors, the molecular weight is greater than 500, and the calculated Log P (CLog P) is greater than 5. Generally, an orally active drug has no more than one violation of these criteria. However, Lipinski specifically states that the Rule of 5 only holds for compounds that are not substrates for active transporters (Lipinski et al., 1997). Silibinin prediction reported no Ro5 criteria violations as reported in Table 4 indicating that the compound has the potential for drug‐like activities.

The results of in silico ADME analysis may be interpreted based on the marginal value compared with resultant value as following: high Caco‐2 permeability is predicted by a value >0.90, and intestinal absorption less than 30% is considered as poorly absorbed; human VDss is low if logVDss is below −0.15 and high if above 0.45; as to BBB permeability, drugs can cross BBB if logBB >0.3, while are poorly distributed if logBB <− 1; as to CNS permeability, drugs with logPS >−2 penetrate CNS whereas those with logPS <−3 are unable to penetrate. Data reported in Table 4 showed that the water solubility value for silibinin was reasonable and revealed respectable absorption estimates, indicating that this drug can be absorbed orally. Silibinin was predicted to be a substrate of P‐glycoprotein as well as P‐glycoprotein I and II inhibitor. VDss prediction supports that silibinin is distributed in tissue rather than plasma.

## 4 DISCUSSION

The hurried spread of SARS‐CoV‐2 has encouraged research to find an existing drug for repurposing scope for a more rapid approach to the pharmacological treatment of this disease. In fact, at that time, a second infection wave is growing and the deaths exceed 2 million. The age, greater than 60 years, and the presence of preexisting comorbidities represent important risk factors with increased COVID‐19 mortality rate, probably due to ineffective immune response versus the viral infection and a greater inflammatory response. In particular, the boosted release of a number of cytokines, called “cytokine storm,” as dysregulated response of the innate immune system to the viral infection, induces fatal damage to lung tissue and/or endothelial dysfunction of micro and macro circulation resulting into systemic thrombosis and associated complications. The efforts to treat COVID‐19 disease are then complicated by the wide‐ranging symptoms and by the many manifestations of virus infection.

For the immediate drug requirement for the treatment SARS‐CoV‐2 infection, active molecules targeting multiple steps in the virus lifecycle are highly wanted. In addition, multiple additional drug effects on cardiovascular system could improve treatment efficacy against COVID‐19 disease.

In this paper, a computational study of the interactions of silibinin with the RBD of the spike glycoprotein and Mpro of SARS‐CoV‐2 was performed. The analysis revealed that silibinin forms a stable complex with RBD so preventing binding to the ACE2 receptor and then virus entry into the host cell. In addition, silibinin showed good negative binding affinity with the Mpro with a very low estimated inhibition constant. Further analysis reported silibinin interactions with many residues on the active site of Mpro supporting the potential effect of this molecule in inhibiting SARS‐CoV‐2 viral replication. These findings are in agreement with those recently reported by Srivastava and coworkers (Srivastava et al., 2020).

It is thought that the primary mechanism by which COVID‐19 causes respiratory failure is pulmonary endothelial dysfunction with diffuse, heterogeneously distributed, pulmonary microthrombi in lungs (Poor et al., 2020), since COVID‐19 irreparably compromise the integrity and the physiological antithrombotic and antiinflammatory properties of the endothelium predisposing to venous and arterial thromboembolic events and worsened outcomes (Abou‐Ismail, Diamond, Kapoor, Arafah, & Nayak, 2020). In fact, the use of heparin was associated with reduced mortality and significantly elevated circulating levels of D‐dimers in patients with severe COVID‐19 infection (Eljilany & Elzouki, 2020; Hsu, Liu, Zayac, Olszewski, & Reagan, 2020). For this reason, many randomized controlled trials on interventions in COVID‐19 targeting endothelial function, are ongoing or planned (Nagele, Haubner, Tanner, Ruschitzka, & Flammer, 2020). In the present study, we further performed a series of experiments focusing on the effects of silibinin on endothelial dysfunction, in order to asses adjunctive therapeutic properties against COVID‐19 disease. In particular, we evaluated the effects of silibinin on proinflammatory and vasoactive factors modulated by TNF‐α in HUVECs. In our experimental conditions, TNF‐α stimulated IL‐6 and MCP‐1 gene expression, inducing an inflammatory condition. Conversely, silibinin pretreatment reduced significantly proinflammatory genes in a dose‐dependent way.

The relationship between endothelial dysfunction and subsequent thrombotic events is already well known in cardiovascular diseases. Endothelial cells functions include maintenance of vascular tone and of a thromboresistant surface among the others, and vasoactive and procoagulant factors play an important role in pathophysiology of endothelium. In particular, the serine protease inhibitor PAI‐1, secreted by vascular endothelial cells, is a critical factor in coagulopathy and thrombosis, since it serves to neutralize the fibrinolytic activity of tissue‐type plasminogen activator (tPA), (Yamamoto, Takeshita, Kojima, Takamatsu, & Saito, 2005). Given that inhibition of PAI‐1 augments the activities of the endogenous plasminogen activators, PAI‐1 has been recognized as a potential therapeutic target for fibrinolytic treatment of thrombotic disorders (Vaughan, 2011).

In hospitalized COVID‐19 patients, PAI‐1 plasma levels were significantly elevated (Goshua et al., 2020) and PAI‐1 mRNA levels are higher in the lungs of COVID‐19 patients, as compared to those of uninfected or influenza patients (Ackermann et al., 2020). Moreover, serum IL‐6 level is positively correlated with the serum levels of PAI‐1 in patients with cytokine release syndrome (CRS), including those with sepsis or ARDS, and the IL‐6 trans‐signaling–PAI‐1 axis is also critical for the pathogenesis of COVID‐19‐induced CRS, which leads to endotheliopathy and coagulopathy (Kang et al., 2020).

In our experimental model, TNF‐α induced PAI‐1, shifting the vascular equilibrium toward a more procoagulant state. Interestingly, silibinin pretreatment reduced PAI‐1 gene expression restoring mRNA levels to baseline, supporting the modulatory activity of this drug on fibrinolytic system in endothelial cells.

Furthermore, endothelial cells produce endothelins, such as ET‐1, that are active vasoconstrictors and may be involved in hemostatic vasoconstriction. Kaffarnik and coworkers (Kaffarnik et al., 2017) have found that increased levels of ET‐1 are a pathological factor in sepsis and contribute to the pro‐inflammatory response. In addition, ET‐1 could have an important role in COVID‐19 thromboembolic events since higher plasma levels of ET‐1 were found in patients with disseminated intravascular coagulation and are considered a predictive factor of poor outcome (Asakura et al., 1992).

Our data demonstrated that cytokine‐induced ET‐1 mRNA overexpression was reverted by silibinin treatment in HUVECs. These effects are in agreement with the recent hypothesis to use bosentan, a dual endothelin‐receptor antagonist approved for the treatment of pulmonary arterial hypertension, in the treatment of SARS‐CoV‐2 (Javor & Salsano, 2020).

In conclusion, these data all support the hypothesis of repurposing silibinin for the prevention and treatment of COVID‐19. In silico data predicted the possibility for silibinin to avoid SARS‐CoV‐2 entry and replication into the host cells. In addition, an advantage is represented by the fact that silibinin pharmacokinetic issues and safety are well known due to its long use for treatment of liver disease (Soleimani, Delghandi, Moallem, & Karimi, 2019). Due to its endothelium antiinflammatory and anticoagulant properties and the capability to interact with SARS‐CoV‐2 main target proteins, silibinin could be a strong candidate for COVID‐19 management from a multitarget perspective. In particular, the employment of silibinin could be particularly relevant for vulnerable patients with preexisting endothelial dysfunction, which is associated with male sex, smoking, hypertension, diabetes, obesity, and established cardiovascular disease, all factors related to adverse outcomes in COVID‐19.

## CONFLICTS OF INTEREST

The authors declare no conflict of interest.

## Data Availability

The data that support the findings of this study are available on reasonable request from the corresponding author, FC.
